# Photophoretic MoS_2_–Fe_2_O_3_ Piranha Micromotors for Collective Dynamic Microplastics Removal

**DOI:** 10.1021/acsami.4c06672

**Published:** 2024-08-27

**Authors:** Víctor de la Asunción-Nadal, Enrique Solano, Beatriz Jurado-Sánchez, Alberto Escarpa

**Affiliations:** † Department of Analytical Chemistry, Physical Chemistry, and Chemical Engineering, Universidad de Alcala, Alcala de Henares, E-28802 Madrid, Spain; ‡ Chemical Research Institute “Andres M. Del Río”, Universidad de Alcala, Alcala de Henares, E-28802 Madrid, Spain

**Keywords:** motion, light, photophoresis, microplastic, degradation, capture, remediation

## Abstract

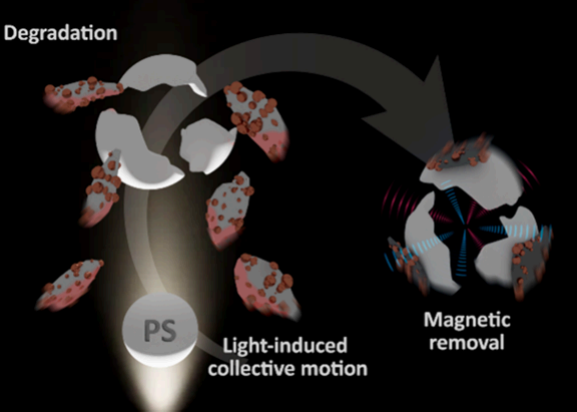

Microplastics are highly persistent emerging pollutants that are widely distributed in the environment. We report the use of MoS_2_@Fe_2_O_3_ core–shell micromotors prepared by a hydrothermal approach to explore the degradation of plastic microparticles. Polystyrene was chosen as the model plastic due to its wide distribution and resistance to degradation using current approaches. Micromotors show photophoretic-based motion at speeds of up to 6 mm s^–1^ and schooling behavior under full solar light spectra irradiation without the need for fuel or surfactants. During this impressive collective behavior, reactive oxygen species (ROS) are generated because of the semiconducting nature of the MoS_2_. Degradation of polystyrene beads is observed after 4 h irradiation because of the synergistic effect of ROS production and localized heat generation. The MoS_2_@Fe_2_O_3_ micromotors possess magnetic properties, which allow further cleaning and removal to be carried out after irradiation through magnetic pulling. The new micromotors hold considerable promise for full-scale treatment applications, only limited by our imagination.

## Introduction

Microplastics (MPs) are small fragments produced by the degradation of plastic wastes into small particles with sizes ranging from 5 mm to 1 μm. In terms of plastic waste generation, in 2020, the production reached 367 Mt worldwide. Notably, the most widely used materials were thermoplastics, including polyethylene, polypropylene, polystyrene (PS), poly(vinyl chloride), and poly(ethylene terephthalate).^1^ Due to their widespread distribution in the environment, along with their chemical stability (i.e., resistance to photodegradation), MPs can be considered as persistent pollutants.^2−4^ Indeed, recent studies indicate that small plastics can affect wildlife, as they can serve as a carrier for pollutants such as heavy metals, pharmaceuticals, personal care products, etc.^5^ The main intake routes to humans and wildlife are ingestion and inhalation.^6^ Furthermore, smaller-sized MPs display a potential cell uptake ability, inducing cytotoxic and genotoxic effects. Still, studies on the specific toxicity of microplastics ought to be performed.^7,8^ Considerable efforts have been made toward the development of highly efficient strategies for the removal of MPs from urban wastewater and the environment, including sorptive removal, electrochemical degradation, advanced oxidation, photodegradation, and biological degradation.^9,10^

Self-propelled nanomaterials such as actuators and micromotors are particularly attractive for addressing the rising concerns on MPs pollution, combining autonomous movement and enhanced fluid mixing with well-established degradation or sorptive removal approaches for highly efficient environmental degradation.^11−15^ For example, a light-responsive hydrogel actuator based on poly(*N*-isopropylacrylamide) was reported as a NIR light-propelled platform for the absorption of MPs by interfacial interactions.^16^ Photocatalytic Au@Ni@TiO_2_ Janus micromotors can assemble via magnetic interactions and be propelled in either water or solutions containing 0.1% H_2_O_2_ for microplastic removal by shoveling.^17^ Light-activated self-electrophoretic hematite/Pt micromotors propelled in 0.1% H_2_O_2_ allow for ROS-mediated MPs degradation.^18^ Alternatively, catalytic core–shell Janus Fe_2_O_3_–MnO_2_ can isolate MPs from water by adsorptive bubble separation in the presence of 5% H_2_O_2_.^19^ TiO_2_ micromotors combine light-induced trapping/degradation of PS nanoparticles, but high levels of peroxide are still required.^20,21^ To avoid the use of toxic peroxide fuel and surfactants, multilayered γ-Fe_2_O_3_/Pt/TiO_2_ micromotors go one step ahead to propel in water by negative photogravitaxis. These micromotors were successfully used for simultaneous adsorptive removal of 50 nm carboxylated PS particles.^22^ In a further study, the ability of the micromotors to generate ROS for poly(ethylene glycol) degradation was exploited. Yet, for adequate degradation (negligible degradation rates were found in pure water), 0.1% H_2_O_2_ was required to improve radical product generation, bringing again the concerns of the use of toxic fuels.^23^ Magnetic algae micromotors, prepared by coating Fe_3_O_4_ nanoparticles with *Chlorella vulgaris*, can interact with micro- and nanoplastics via electrostatic interactions (imparted by the −COOH groups of the algae), reaching 92% removal efficiencies.^24^ Still, the limited speed of the magnetic-based micromotors (16–35 μm/s) can hinder their applicability for realistic treatment of highly polluted water.

Apart from sorptive removal, degradation approaches have been exploited for micromotor-based MPs degradation. For example, magnetic polydopamine@Fe_3_O_4_ micromotors modified with lipase can degrade polycaprolactone (PCL) MPs. As a drawback, the required time for successfully degrading the MPs is up to 24 h.^25^ Photocatalytic micromotor-based approaches comprising BiVO_4_/Fe_2_O_3,_^26^ BiOI-Fe_3_O_4_,^27^ or hematite/metal micromotors^28^ driven by self-electrophoretic mechanisms under light irradiation can efficiently remove poly(lactic acid) and PCL as model MPs. Still, the self-electrophoretic propulsion mechanism requires at least 0.1% peroxide fuel for propulsion, limiting the full-scale applicability of such strategies. Recent trends in the field are aimed at the combination of magnetic navigation-MPs trapping, followed by photocatalytic degradation. Thus, antimony sulfide/ferrite^29^ or Fe_3_O_4_@BiVO_4_^30^ micromotors have been applied for MPs capture, followed by UV light irradiation for ROS generation and degradation. While the micromotor motion is improved, in some cases, peroxide is still needed for enough ROS production toward MPs degradation.

Our group has illustrated the light-driven photophoretic motion of transition metal dichalcogenide (TMD) microflakes prepared by exfoliation of the pristine material. The micromotors can propel by just vis light irradiation, generating ROS and a highly efficient movement (reaching speeds of 12 mm/s), which can be exploited for MPs degradation.^31^ Inspired by the previous micromotor approaches and our previous findings on TMD-based ROS production, herein we report MoS_2_@Fe_2_O_3_ core–shell micromotors for on-the-move degradation and trapping of MPs. The micromotors are prepared by a hydrothermal approach, resulting in a synergetic entity combining the photothermal abilities of MoS_2_ and the magnetic properties of Fe_2_O_3_ nanoparticles (NPs). Owing to their enhanced photothermal conversion capabilities, TMD-based micromotors show highly efficient photophoretic propulsion under UV and visible irradiation. Furthermore, the magnetic properties of the Fe_2_O_3_ NPs allow for their magnetic actuation and removal. Remarkably, photophoretic micromotors display efficient propulsion without the requirements of fuel or surfactants. Additionally, the semiconducting nature of MoS_2_ in connection with Fe_2_O_3_ NPs will be exploited for the dramatically enhanced generation of ROS (as compared with the use of individual materials), ultimately aiming for the degradation of MPs.^32,33^ As such, the MoS_2_@Fe_2_O_3_ micromotors can compromise the structural stability of 20 μm PS microbeads due to a synergistic effect of ROS production, localized heat generation, and physical scarring without the need for additional reagents. Please note that the model used (PS, high density, not expanded) has been chosen for its significance, but its degradation is very hard to obtain. Compared with previous micromotor works, the use of hydrogen peroxide is avoided, along with highly remarkable speeds to propel even in highly contaminated samples. Finally, due to the magnetic properties of the synthesized microcomposites, further cleaning and removal can be performed after the irradiation through magnetic pulling.

## Experimental Section

### Reagents and Materials

Molybdenum disulfide (cat. 234842), (NH_4_)_2_MoS_4_ (cat. 323446), hydrazine (cat. 309400), Fe_2_O_3_ nanoparticles (cat. 544884), and PS particles (20 μm, cat. 74491) were purchased from Merck (Madrid, Spain) and used as received without further purification. All solutions were prepared using ultrapure water (18.2 MΩ cm resistivity at 25 °C).

### Synthesis of MoS_2_ Micromotors

A 0.75 mg/mL sample of MoS_2_ in ultrapure water was placed in a vial and sonicated using a tip sonicator (ultrasonic processor VCX 130, Vibra-cell Sonics) for 1 h or until the flakes displayed phototaxis.

### Synthesis of MoS_2_@Fe_2_O_3_ Micromotors

(NH_4_)_2_MoS_4_ (35 mg) was mixed with 4 mg of Fe_2_O_3_, followed by addition of 20 mL of ultrapure water. The mixture was sonicated for 10 min using the tip sonicator until a brown solution was obtained. Next, the sample was placed in an autoclave, followed by the addition of 31.6 μL of hydrazine. The autoclave was introduced in the stove at 200 °C for 8 h. The resulting solution was centrifuged for 5 min at 5000 rpm. The isolated solid was redispersed in 20 mL of ultrapure water.

### Characterization of the Micromotors

A Jeol JSM 6335F scanning electron microscope was used to characterize the micromotors, using an acceleration voltage of 15 kV. The EDX mapping analysis to obtain a map of the elemental composition of the microtubes was carried out using an Oxford Instruments X-Max, with a resolution of 127 eV–6 keV. Transmission electron microscopy (TEM) images were taken using a Zeiss M-10 microscope. Raman characterization was performed using Alpha300R-Alpha300A AFM Witec equipment. A Zetasizer Nano ZS (Malvern Panalytical, United Kingdom) was used to measure the zeta potential, using Malvern Zetasizer software to treat the data. Measurements were performed at 25 °C, 1.6 index refraction, 0.01 absorption, and pH 7.

### Micromotor Movement and Degradation Experiments

An inverted Nikon Eclipse Instrument Inc. Ti-S/L100 optical microscope, coupled with a Zyla sCMOS camera and 20× and 40× objectives, was used to capture videos. The microscope was equipped with a xenon arc lamp light source to promote micromotor movement and to perform the degradation experiment. The light source indices directly on a drop or a vial containing the micromotors were inserted through the microscope objective. The energy output was measured by using a Thorlabs optical power meter (PM100D).

FTIR analysis during the degradation processes was performed using an FTIR Bruker IFS66 V. MALDI-TOF analysis was conducted with a Bruker ULTRAFLEX III TOF/TOF. For the analysis, the aqueous extracts containing the PS particles and the micromotors were evaporated with N_2_ to dryness and redissolved in 250 μL of methanol. Next, the samples were mixed with 10 mg/mL of *trans*-2-[3-(4-*tert*-butylphenyl)-2-methyl-2 propenylidene] malononitrile (DCTB).

### Calculation of Maximum Mechanical Pressure on a Single Impact

To estimate the pressure of the micromotors onto the microparticles’ surface, the micromotors were assumed as 1 × 1 × 0.2 μm rectangular prisms. The mass of each micromotor was estimated by assuming the density of pure MoS_2_ (5.06 g cm^–3^). The kinetic energy was estimated as *E* = 0.5 × *m* × *v*^2^ (1.5 × 10^–19^ J), and the impact force was calculated as (*F* = *E*/*d*) (1.6 × 10^–11^ N) with the displacement of a single collision assumed as 1% of the micromotor length (maximum force scenario). Finally, the mechanical pressure was obtained by dividing the impact force and the micromotor section by 0.2 μm^2^ and given in units of 80 N m^–2^.

### Statistical Analysis

All the data presented (unless stated otherwise) are presented as the mean ± SD of *n* = 3 analysis. Origin lab software was used for statistical analysis and data display.

## Results and Discussion

**Figure 1:**
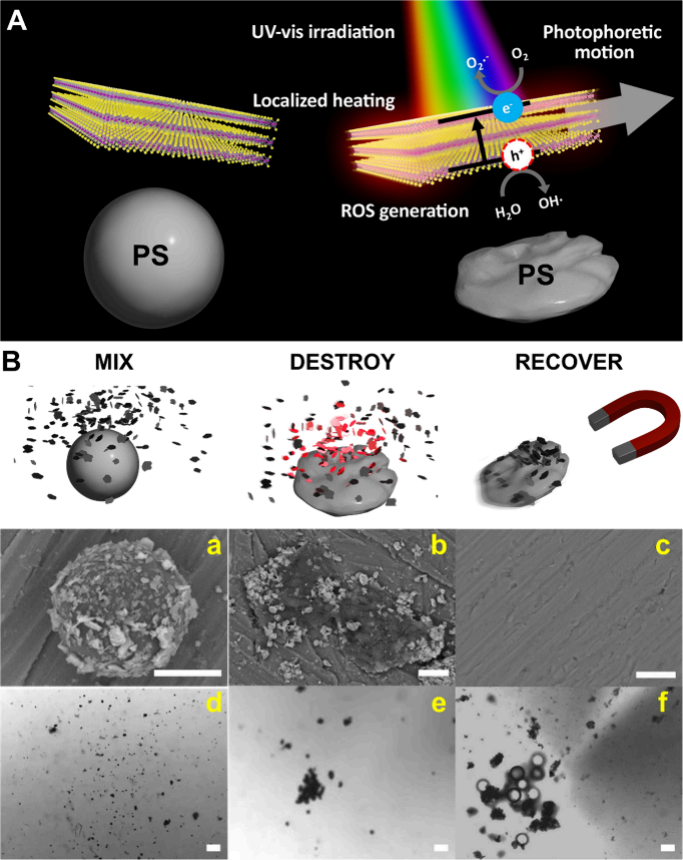
(A) Schematic representation of light-induced MPs degradation and motion of photophoretic MoS_2_@Fe_2_O_3_ micromotors. (B) Schematic of the removal process and corresponding SEM and microscopy image figures before (a) and after (b) irradiation and removal (c) of PS beads with the MoS_2_@Fe_2_O_3_ micromotors. Swarming motion of MoS_2_@Fe_2_O_3_ micromotors before (d), during (e), and after (f) irradiation (taken from Video S1). Please note that in the video, the captured images were taken right after the micromotor contact with the PS beads before conducting any degradation experiment. Scale bars: 20 μm.

Figure 1A illustrates the concept of photophoretic MoS_2_-based micromotors for MPs removal. The micromotors display inherent photothermal behavior and can heat up upon light irradiation. Such heat is released into the medium, promoting a hydrodynamic flow responsible for the fast movement of the micromotors and swarming behavior, at speeds of up to 6 mm s^–1^. In addition, the micromotors are based on photocatalytic MoS_2_, displaying inherent electronic levels with an experimental direct bandgap of 2.6 eV. When the incident irradiation energy is higher than such a bandgap, the promotion of electrons from the valence to the conduction band is allowed. Indeed, the positive electronic holes and the conduction electrons in the photocatalyst are hot spots for the reaction with the water media and dissolved oxygen, generating ROS for MPs degradation.^31^ As such, and as can be seen in Figure 1B, the mechanism for MPs morphological degradation (mix and destroy) and subsequent removal relies on the rapid swarming behavior of the micromotors under irradiation with UV–vis electromagnetic radiation (full light spectra). The fast motion of the micromotors allows them to attach to the PS particles’ surfaces. Once the particles are fixed on the MPs, a localized temperature increase (heat transfer to MPs) along with ROS generation is responsible for the synergetic degradation of PS. Furthermore, magnetic pulling can be used for the removal of both the micromotors and remaining degraded MPs.

**Figure 2:**
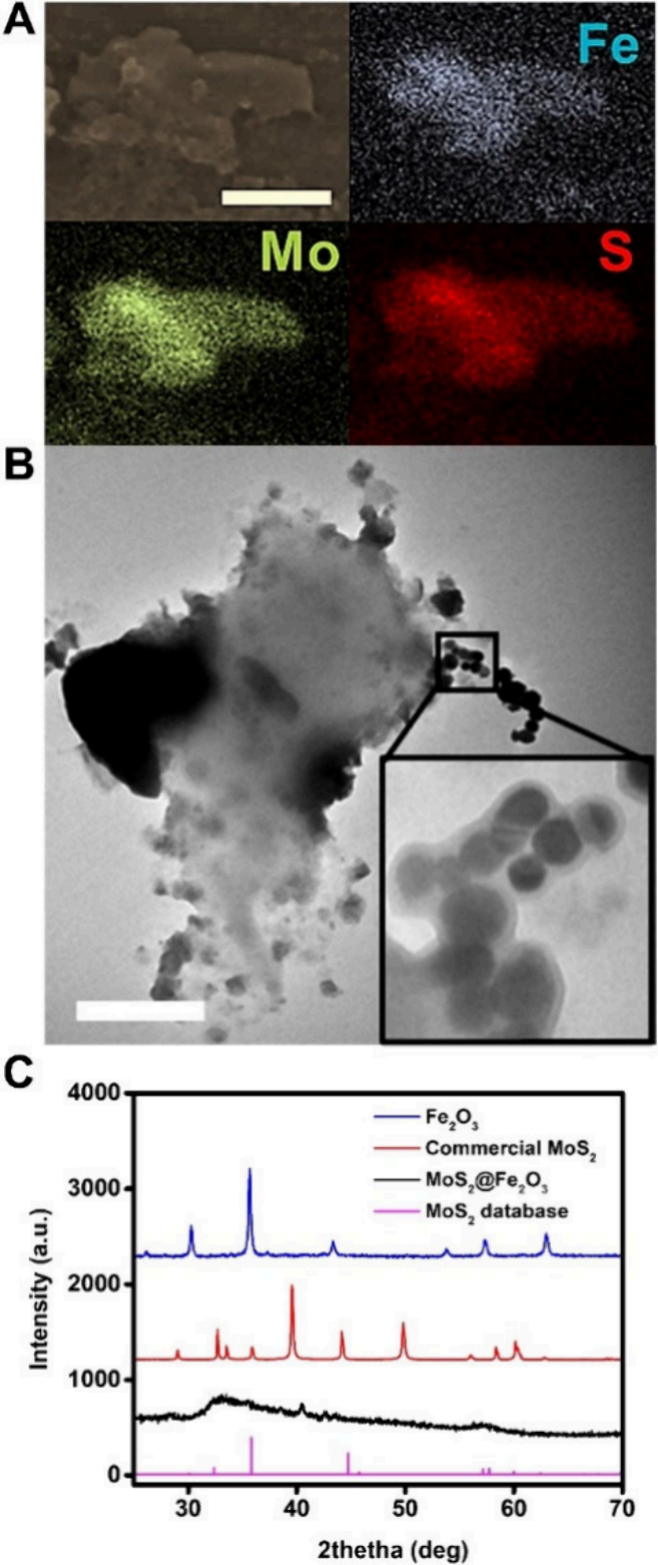
Characterization of the hydrothermal MoS_2_@Fe_2_O_3_ micromotors. (A) SEM images and corresponding EDX mapping. (B) TEM images of micromotors. (C) X-ray diffraction patterns of Fe_2_O_3_ nanoparticles, commercial MoS_2_ control micromotors, MoS_2_@Fe_2_O_3_ micromotors, and MoS_2_ reference spectra from the International Centre for Diffraction Data (ICDD: 01-086-3467) database. Scale bars: 500 nm.

Successful micromotor synthesis is key for obtaining highly efficient MPs removal. The magnetic MoS_2_@Fe_2_O_3_ micromotors were synthesized by a hydrothermal approach in a Teflon-lined stainless-steel autoclave (for more details, see the Experimental Section). In this case, instead of exfoliation of pristine MoS_2_, we use its precursor [(NH_4_)_2_MoS_4_], which is mixed with Fe_2_O_3_ nanoparticles, followed by sonication to promote its interaction. Next, the dispersion is mixed with hydrazine in an autoclave at 200 °C (8 h) to promote the reduction of (NH_4_)_2_MoS_4_ into MoS_2_.^34,35^ For comparison and to get insights into the influence of the change in the synthetic mechanism and role of Fe_2_O_3_ NPs in the ROS generation and micromotor propulsion, control MoS_2_ micromotors were synthesized by controlled exfoliation of the bulk material in water. As can be seen in Figure 2, the hydrothermal approach results in the generation of microflakes/micromotors with sizes ranging from 360 ± 120 nm. In the case of exfoliated MoS_2_, the size is 500 ± 200 nm (see Figure S1 in the Supporting Information). Furthermore, a uniform element distribution is illustrated in the energy-dispersive X-ray spectroscopy (EDX) images in Figure 2A. Moreover, transmission electron microscopy (TEM) characterization further supports the amorphous morphology and presence of edges, along with the successful incorporation of the Fe_2_O_3_ nanoparticles (see Figure 2B).

**Figure 3:**
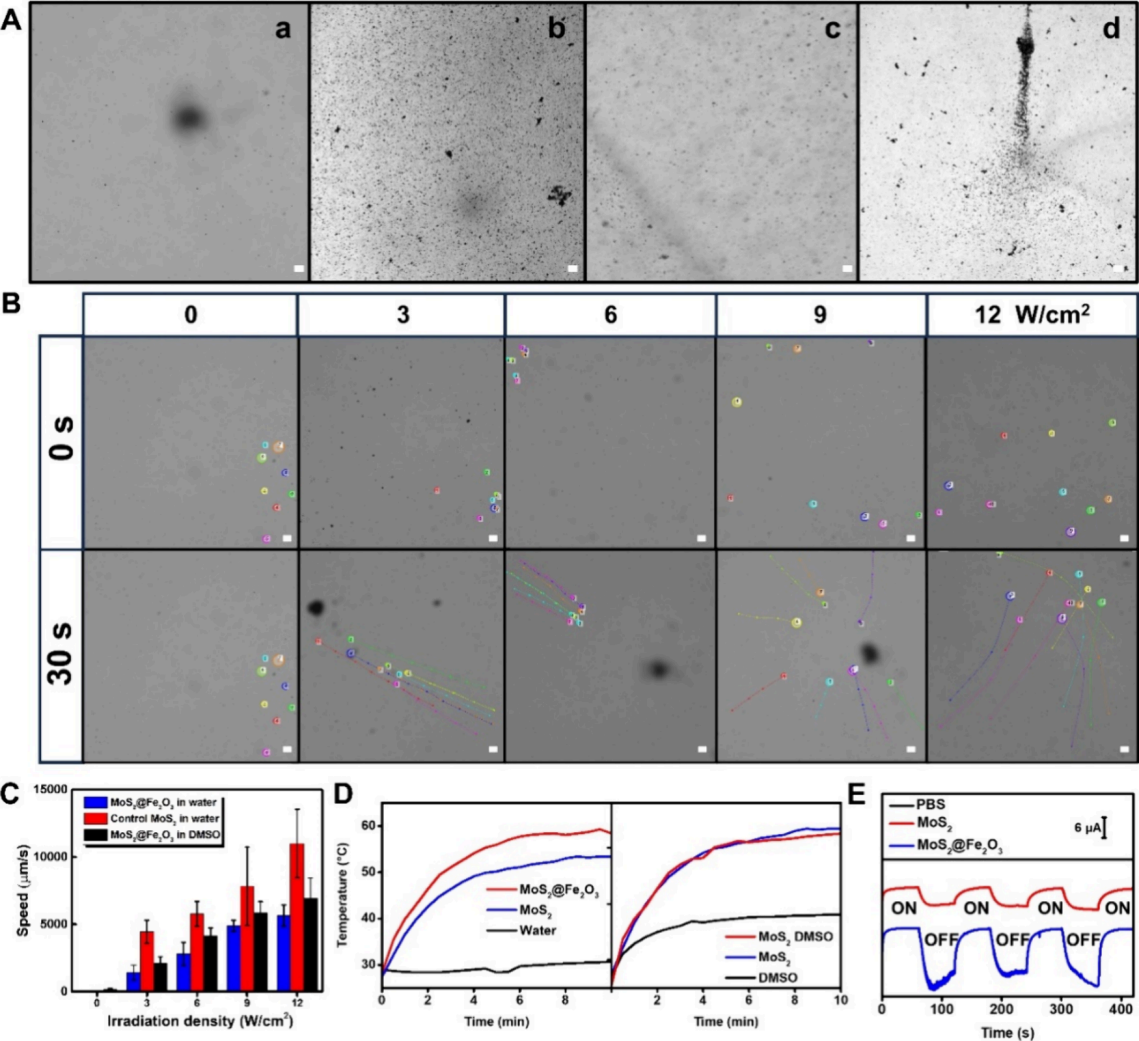
Characterization of the motion of the MoS_2_@Fe_2_O_3_ micromotors. (A) Time-lapse images (taken from Video S2) of the motion of MoS_2_@Fe_2_O_3_ solutions under (a) and in the absence of (b) UV–vis irradiation in water; MoS_2_@Fe_2_O_3_ micromotors in DMSO (c) and MoS_2_ control micromotors in water (d). (B) Time-lapse images (taken from Video S3) of the propulsion and representative motion trajectories of the MoS_2_@Fe_2_O_3_ micromotors prior to (0 s) and after (30 s) UV–vis irradiation in water. (C) Speed profiles of the MoS_2_@Fe_2_O_3_ micromotors under different irradiation densities and media (water and DMSO). For comparison, the speeds of control MoS_2_ micromotors are also included. (D) Time-dependent temperature recording of water and MoS_2_ and MoS_2_@Fe_2_O_3_ solutions under UV–vis irradiation. (E) Chronoamperometry profiles of PBS supporting electrolyte, MoS_2_ micromotors, and MoS_2_@Fe_2_O_3_ micromotors in subsequent cycles of ON-OFF UV–vis light irradiation. Scale bars, 20 μm. Error bars represent the standard deviation of 10 measurements.

The scanning electron microscopy (SEM) images of Figure 1B illustrate the attachment of the micromotors to the PS particles before (a) and after (b) irradiation with light (b). Notably, the micromotors rapidly coat the PS microbeads. Once attached, the micromotors heat up generating hot-spots on the surface of the nanoparticles. According to preliminary research, transition metal dichalcogenides can generate hot-spots of up to 500 °C when irradiated with high-intensity light sources.^31^ Due to heat dissipation from the hot-spots, the micromotors heat the bulk solution to 60 °C (see later studies shown in Figure 3D), which along with localized ROS production (Figure 3E) results in the degradation of the PS spheres in a time-dependent manner (Figure 1B, b).

It is possible to tailor the content of Fe_2_O_3_ NPs by hydrothermal synthesis using a magnetic iron NP as a seeding agent. Indeed, this synthetic process grants photocatalytic microcomposites with magnetic properties. As such, magnetic pulling can be exploited to remove both the micromotors and the remaining MP particles (Figure 1B, c). This is further illustrated by the time-lapse images of Figure 1B, d–f (taken from Video S1) which illustrate the uniform distribution of the micromotors in the solution before light irradiation without appreciable interaction with the PS particles. Under irradiation, a rapid swarming behavior is noted, generating collisions between the micromotors and the PS particles in the solution for subsequent degradation. It should be noticed here the highly efficient operation of the micromotors in the absence of toxic peroxide fuel or surfactant, as compared with previous approaches, and with a remarkable speed that avoids hampered operation in real media.

In further XRD characterization (see Figure 2C), commercial Fe_2_O_3_ was identified, and the main peaks correspond to those previously reported for the maghemite phase in the bibliography. Namely, the (220) peak appeared at approximately 30°, the (311) being the most intense diffraction peak appeared at approximately 36°, and lower intensity peaks were located at 43°, 54°, 57°, and 63° corresponding to the (400), (422), (511), and (440) peaks, respectively.^36^ Next, commercial bulk MoS_2_ was studied; accordingly, the main bands (004), (100), (103), (006), and (105) were located at 29°, 33°, 39°, 44°, and 50°, approximately. This corresponds to previously reported XRD patterns of bulk MoS_2_.^37^ Finally, the XRD pattern of MoS_2_@Fe_2_O_3_ was recorded. Notably, the pattern of the composite corresponds to that of commercial MoS_2_. This indicates the nanostructuring of MoS_2_ in the hydrothermally synthesized micromotors. As such, the main peaks (101) and (110) are located at roughly 32° and 57° as previously reported.^38^ For comparison, a MoS_2_ reference diffraction pattern was included (ICDD 01-086-3467). Notably, the diffraction pattern of the as-synthesized MoS_2_@Fe_2_O_3_ composite does not show the main peaks of Fe_2_O_3_. This may indicate a low content of maghemite nanoparticles in the structures of the micromotors. Nonetheless, both TEM images and EDX mapping show a good distribution of magnetic nanoparticles throughout the micromotor structure. Relevantly, there is a remarkable difference between the bulk and nanostructured MoS_2_ diffraction patterns as reported.^37,38^ Summarizing, the MoS_2_@Fe_2_O_3_ shows an overall amorphous structure with evenly distributed Fe_2_O_3_ nanoparticles inside the MoS_2_ as the results from TEM and EDX mapping suggest. Furthermore, the broadening of the main peaks is observed due to the lower average crystallite size.

Once the micromotors were characterized, and before studying the performance for MPs degradation and removal, we characterized the motion behavior of the hydrothermal micromotors. As can be seen in Figure 3 A, a and Video S2, the MoS_2_@Fe_2_O_3_ micromotors experience a fast movement and swarming toward a local point after irradiation with the full light spectra. No movement is noted in the absence of light, and the particles exhibit the typical Brownian motion (Figure 3A, b). Similar moving behavior is observed for the control MoS_2_ micromotor (Figure 3A, d) prepared by exfoliation, indicating that the chosen synthetic route has a negligible effect on the photophoretic properties of the micromotors. Our research group has previously illustrated that semiconductor materials such as WS_2_ or MoS_2_ display nonradiative relaxation, which leads to the unique phenomena of an increase in the lattice temperature or the generation of temperature gradients.^31,39,40^ The heat dissipation in the solvent (in this case, water) generates a gradient in the form of a directional, hydrodynamic flow toward the light spot, or positive photophoresis. As a result, a photophoretic micromotor swarm is generated.^41^ In this work, we employed MoS_2_ as the base material for decoration with magnetic Fe_2_O_3_ NPs for magnetic control. Please note here as well that such nanoparticles possess also photothermal properties, which can play an active role in photophoretic propulsion as well. Also, as depicted by the red-shift on the Fe_2_O_3_@MoS_2_ composite compared to MoS_2_ (Δ*E* = −0.8 eV, Figure S2), it may influence the overall efficiency, allowing for the use of a wider region of the UV–vis spectra for the generation of heat and ROS.

First of all, we checked if the propulsion mechanism can be attributed to self-diffusiophoresis (due to the photocatalytic activity) or photophoretic effects (due to the photothermal capabilities of MoS_2_ and Fe_2_O_3_).^42^ To this end, we checked the propulsion in water (Figure 3 A, a) and in dimethyl sulfoxide (DMSO, Figure 3 A, c) as a nonionizable solvent to prevent the generation of ROS from water, thus limiting the self-diffusiophoretic motion mechanism. The micromotors move at similar speeds in both media, indicating that the diffusiophoretic mechanism is not responsible for the observed motion. Therefore, the motion of the micromotors can be attributed to photothermal effects, namely, due to the photophoresis mechanism. The interaction of incident radiation with the electronic structure of the MoS_2_ generates a localized heating, which during dissipation renders a hydrodynamic flow responsible for the collective micromotor behavior.^41,43^ The motion behavior can be controlled by regulation of the irradiation intensity, as reflected in the time-lapse images and tracking trajectories in Figure 3B and Video S3. As can be seen, as the intensity of the incident light increases, the micromotors exhibit a marked swarming effect, positive photophoretic motion toward the incident light, with a linear increase in the speed (see Figure 3C), reaching velocities of up to 5600 ± 790 μm/s, which correspond to an irradiation density of 12 W/cm^2^ (the power output was measured with an optical power meter, and the power density was estimated by taking into consideration the irradiated cross section).

As can also be seen in Figure 3C, a similar trend in the speed is observed in DMSO media, which further supports our observations and conclusions on the motion mechanism. Regarding the speeds, the incorporation of the magnetic materials results in a slight reduction to 4 mm/s compared with the 12 mm/s of MoS_2_ (see Figure 3C). This fact, however, does not hamper future practical applications, as the speed of the micromotors is remarkably high. This can be probably due to the different synthetic routes used.

To check the role of temperature on propulsion and get further insights into the propulsion mechanism, we recorded the temperature in water and solutions containing the micromotors during light irradiation at different times using a thermocouple (Figure 3D). After 10 min, the temperature increases up to 60 °C, which is more noticeable in the case of MoS_2_@Fe_2_O_3_, due to the synergistic effect of Fe_2_O_3_, which is also a photothermal material.^43^ Please note that in water (running as control) the temperature remains unaltered; thus, such an increase is associated with the inherent photothermal properties of the micromotors. This effect is also observed in DMSO (with the sole exception of the slight temperature increase of the DMSO control solution), demonstrating the temperature-dependent nature of the photophoretic mechanism. Finally, the production of ROS was studied by amperometry using an ITO electrode and a portable potentiostat. The electrode was placed on top of the microscope objective, followed by dropping 50 μL of the MoS_2_@Fe_2_O_3_ or MoS_2_ micromotors. The amperometric curves of subsequent cycles of ON–OFF radiation in water and solutions containing the micromotors of Figure 3E indicate radical production during irradiation, with a decrease in the signal when irradiation is stopped. This contrasts with the stable profile in water samples. Additionally, to get further insights into the amount of radical produced, we performed a calibration plot adding increased concentrations of H_2_O_2_ and measuring the variation of the amperometric signal. The results are plotted in Figure S3. Next, we extrapolated the signal obtained from the irradiation of the micromotors and extrapolated it in the calibration plot. The signal corresponds to a concentration of 81 and 32 mM of H_2_O_2_ of radical production with MoS_2_@Fe_2_O_3_ and MoS_2,_ respectively. Thus, the experimental data support the role of swarming movement and attachment to PS, heating, and ROS production on PS particle degradation.

**Figure 4:**
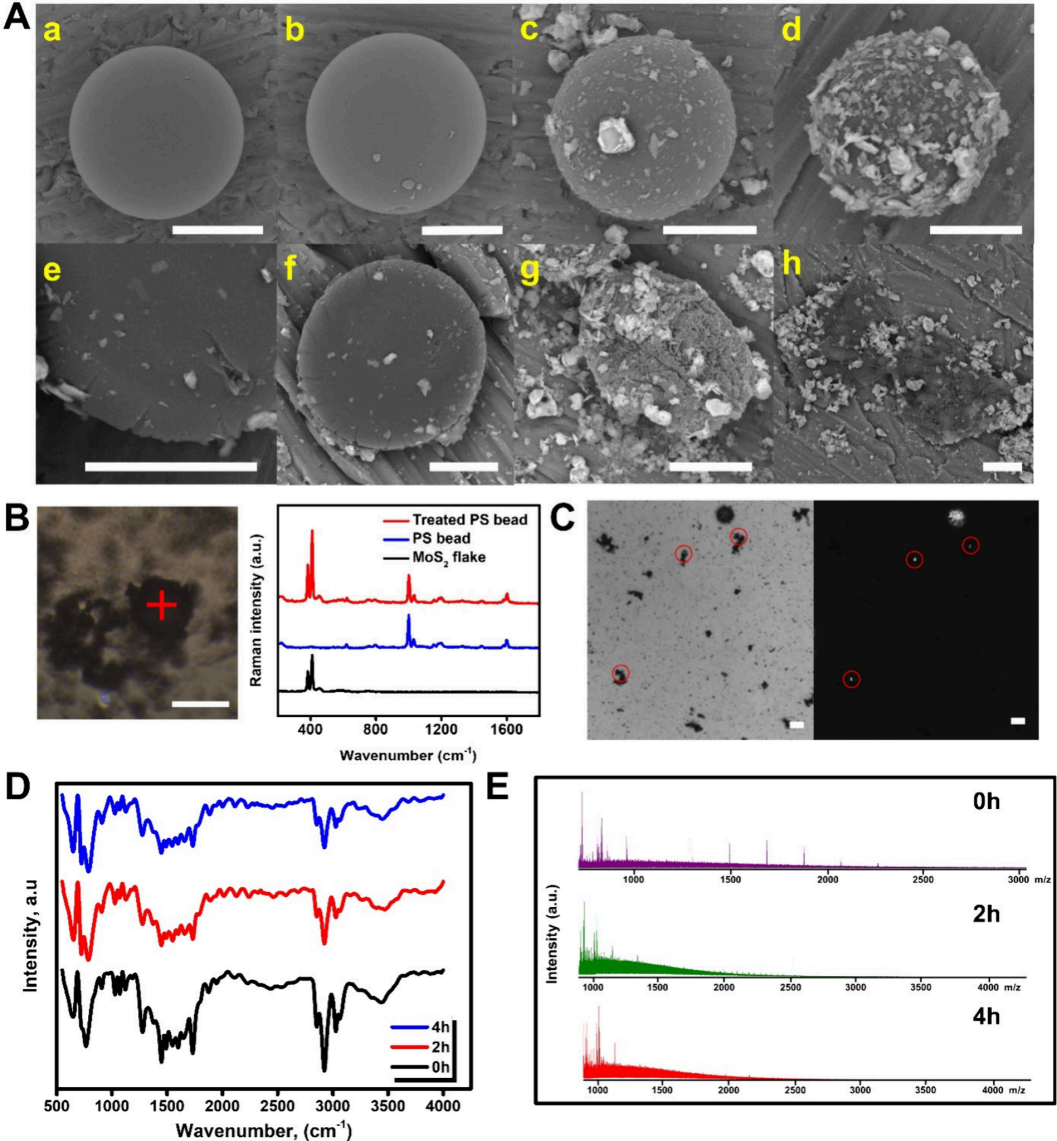
MPs removal and control experiments. (A) SEM images of PS beads in water under UV–vis irradiation (a), PS beads in DMSO under stirring without irradiation (b), PS beads with the micromotors under UV–vis irradiation in DMSO (c), and PS beads with the micromotors under stirring without UV–vis irradiation in water (d). Morphological degradation experiments of PS beads were performed in water with the micromotors under UV–vis irradiation. Sample volume = 1 mL. Exposure time: 30 min (e), 1 h (f), 2 h (g), and 4 h (h). Scale bars: 10 μm. (B) Confocal optical microscopy image of treated PS beads and corresponding Raman spectra of treated and untreated PS beads and the micromotors. (C) Optical microscopy images of fluorescein-labeled PS microbeads and corresponding fluorescence images after treatment with the micromotors. (D) FTIR and MALDI-TOF (E) spectra of the treated samples before and after 2 and 4 h exposure with the micromotors. In B, D, and E, three independent measurements were taken, but one was selected for simplicity. Total irradiation time for degradation experiments: 4 h. Scale bars, 20 μm.

Next, we conducted a series of degradation and control experiments using PS particles as model MPs. For the experiments, the micromotors were mixed with PS particles and irradiated with UV–vis light for 30 min and 1, 2, and 4 h. Control experiments to check the effect of ROS production on degradation were also performed in DMSO. It is well-known that MPs analysis is challenging, with major routes being mass analysis and particle analysis.^1^ In this work, we adopted morphological SEM observation along with Raman, FTIR, and fluorescence labeling to check the potential degradation (Figure 4). The experiments were performed by mixing a dispersion of PS beads with the micromotors, running the appropriate control experiments in parallel. PS was chosen as a model MP as it is one of the most widely used plastics, present in many products (electronics, food containers) but also because of its relatively high chemical inertness and difficult degradation, which require special treatments such as thermal or catalytic pyrolysis under controlled atmosphere.^44,45^

As can be seen in Figure 4A, no apparent degradation is observed after irradiating the PS solutions in water, as testified by the structural integrity of the beads (part a of the figure), indicating that the irradiation by itself does not have any effect on it. In a similar manner, DMSO does not exert any effect on PS beads’ degradation (part b in the figure). Next, two control experiments were performed. First, the PS beads were added to a micromotor dispersion in DMSO and irradiated for 4 h (Figure 4A, c). Second, PS beads were added to a micromotor dispersion in water and stirred for 4 h without UV–vis irradiation (Figure 4A, d). In both cases the morphological integrity of the PS microbeads was maintained, with no apparent degradation. These control experiments indicated, first, the crucial role of ROS production for degradation, considering that ROS are not produced in DMSO or the absence of micromotors irradiation. Hence, we can confirm that irradiation with UV light alone does not trigger the degradation of PS microparticles. Second, physical scarring does not have any effect on degradation, as testified by the entireness of the PS beads in experiments in water under stirring and micromotors moving in DMSO. Furthermore, considering the speed and physical features of the micromotors, the exerted mechanical pressure is less than 80 N m^–2^ per micromotor, which is insufficient for considering a physical disruption of the MP structure. In sum, these observations testified our hypothesis for PS beads degradation with the micromotors: a combination of thermal effects and ROS production, which is enhanced by the schooling and attachment of the micromotors with the PS microparticles, for further magnetic pulling and removal. This fact is supported by the SEM observation of experiments with moving MoS_2_ micromotors in water. Thus, as can be seen in Figure 4 A, e, after 30 min treatment, apparent damage is observed with fissures and an altered morphology in the PS beads. The process progressed, and after 1 h of treatment, the surface of the PS beads was clearly damaged (part f). More structural damages are observed after 2 h, notably deforming the bead from a sphere to an ellipsoid-like structure (part g). Finally, after 4 h treatment, the structural integrity of the beads was severely compromised; as observed, the particle lost its spherical shape and opened into two shell-like flat structures.

Raman observation and fluorescence-labeling studies were performed as an additional test. Figure 4B illustrates the Raman spectra of the treated PS beads, untreated PS beads, and micromotors. The overlapping spectra display both the bands at 370–400 cm^–1^ (indicative of MoS_2_)^46^ and representative bands of the PS: 621 cm^–1^ (ring deformation mode), 795 cm^–1^ (C–H out-of-plane deformation), 1001 cm^–1^ (ring deformation mode), 1031 cm^–1^ (C–H in plane deformation), 1155 cm^–1^ (C–C stretch), 1583 cm^–1^ (C=C stretch), and 1602 cm^–1^ (ring skeletal stretch).^47,48^ After treatment, a decrease of the bands at 1001, 1031, and 1155 cm^–1^, associated with C–C and C–H bonds, is noted. PS is composed by a network connected by strong C–C and C–H bonds. There are two main reported mechanisms for the degradation of PS mediated by ROS (Figure S4). One is termed the C–H oxidation pathway, which relies on the reaction with ROS (O_2_•^–^) to form an alkyl radical intermediate and water. This radical species can further react with dissolved O_2_ to form peroxyl radical intermediates (−COO•). Hydroperoxides (−COO–H) can then be formed by intramolecular H transfer in the polystyrene system, leading to the formation of further radical intermediates and enabling further in situ radical formation. Finally, ROS can induce the formation of alkoxy polymer radicals (−CO•). The C–C bond can then be cleaved by undergoing β-scission to form a carbonyl compound (α) and an alkyl radical (β). In the other mechanism, based on radical elimination, the PS chains can undergo heat-mediated radical elimination after forming a radical intermediate with the ROS. It is worth noting that the radical elimination method requires high temperatures, whereas the C–H oxidation pathway is favored in highly acidic media. Hence, in the reported experimental conditions, the thermal depolymerization of PS induced by the formation of radicals and high temperatures in the micromotors’ hot-spots may lead to the degradation of the polystyrene microbeads whereas the degradation through the formation of peroxyl radical intermediates may be favored at low pH.^49^ The combination of such effects results in the disintegration of the PS microparticles, as also testified in the experiments of Figure 4C, which illustrates the labeled, broken pieces coming from the previously labeled PS microparticles’ prior treatment. The FTIR spectrum of PS particles (see Figure 4D) before treatment reveals the polymer chemical structure, with strong signal at 3000–2780 cm^–1^ associated with the strong C–H bond stretching modes associated with the polymeric backbone (sp^3^ C formation). In the FTIR after 2 and 4 h treatment such signals decrease greatly, indicating that the micromotors are degrading the PS by attacking the polymeric backbone.^50^ Data collection was performed in triplicate; the figure shows one measurement for simplicity.

Next, we try to observe the potential generation of PS degradation products or changes in the initial PS solution profile through the treatment by matrix-assisted laser desorption/ionization (MALDI-TOF) and gas chromatography–mass spectrometry (GC–MS) of extracts of the PS particle solutions treated with the micromotors at the different times tested initially. For the experimental conditions, please see the Experimental Section. The results obtained are given in Figure 4E. In the solution containing PS prior treatment with the micromotors, representative peaks at *m*/*z* 1199, 1330, 1514, 1699, 1883, 2067, and 2252.1 were identified. After 2 and 4 h treatment with the micromotors, the intensity of such peaks decreases, as illustrated by the absence in the MALDI/TOF mass spectrum. The peak intensities are also listed in Table S1. Additionally, to check for potential degradation products, the *m*/*z* range scanned was extended to 4000. Yet, no further degradation was observed, reflecting the complexity of the samples and the difficulties in the analysis of microplastics. From the MALDI/TOF observation, it can be concluded that some degradation of PS is obtained (as also observed in the FTIR) by the action of the micromotors, as reflected by the disappearance of the initial peaks representative of PS.^51−54^ Measurements were performed in triplicate; the figure shows one measurement for simplicity.

**Figure 5:**
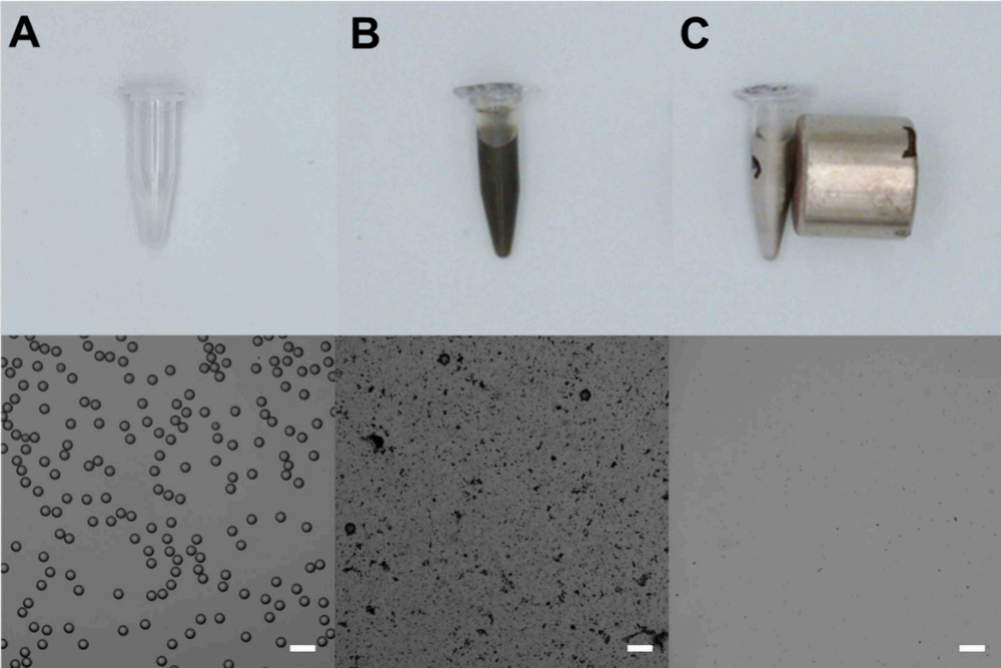
Pictures (top) and optical microscopy images (bottom) of vials containing (A) PS microbeads, (B) PS microbeads after treatment with MoS_2_@Fe_3_O_2_ micromotors, and (C) PS microbeads after irradiation and magnetic pulling. Scale bars, 50 μm.

It should be noted here that similar results were obtained for PS degradation with MoS_2_ and MoS_2_@Fe_2_O_3_ micromotors. Thus, for future cost-effective synthesis and to tailor the application for an intended future approach, both materials can be used alone or at different rates. Yet, as illustrated in Figure 5, the hydrothermal MoS_2_@Fe_3_O_2_ micromotors possess the added advantage of the easy removal of the degradation spots by magnetic pulling. Please note the clear change in the black color of the solution, from deep black (Figure 5B) due to the micromotors to a clear color and the accumulation at one spot due to the magnetic attraction toward the magnet (Figure 5C). This is also further reflected in the corresponding microscopy images before and after magnetic pulling, where the high density of degraded PS and the micromotors practically disappear, indicating a facilitation in the removal after degradation for future recycling.

The zeta potential of both PS microbeads and micromotors was measured to understand the interaction between both materials. PS microbeads are neutral to slightly negatively charged (−6.5 ± 0.8 mV), whereas MoS_2_@Fe_3_O_2_ micromotors display negative surface charge (−20.9 ± 0.8 mV). Notably, due to the lack of π orbitals in the MoS_2_ structure, similar surface charge, and the poor reactivity of polystyrene, the interaction between the micromotors and the remaining microplastic particles is expected to happen through weak physical nonspecific interactions followed by high-speed collisions.

Summarizing, UV–vis irradiation leads to a fast convective motion that in turn induces several effects: (1) fast photophoretic motion of the MoS_2_@Fe_2_O_3_ micromotors, (2) physical interaction between the micromotors and PS microbeads, (3) capture of microplastics and trapping in the focal point (see Video S1), (4) heating of MoS_2_@Fe_2_O_3_ micromotors inducing the generation of hot-spots and heating transfer to the MPs, and (5) in situ generation of ROS. The combination of these effects leads to the morphological degradation of the PS as MPs and capture of the MPs (mainly due to physical interaction promoted by high-speed collisions). The plastic particles can be then removed by magnetically actuating the physically attached micromotors. Compared with recent literature works (see Table S2), our approach combines a new material with different functions for degradation of microplastics as illustrated with PS as a model.

## Conclusions

We have reported the application of magnetic core–shell MoS_2_@Fe_2_O_3_ micromotors for highly efficient PS removal as a model MP. Degradation is achieved by a synergistic mechanism comprising photophoretic micromotor motion, attachment to the particles, and temperature-increase/ROS generation for the disintegration of the PS particles after 4 h of treatment. The introduction of magnetic nanoparticles allows for the removal of the resulting pieces by magnetic pulling. Unlike the previously developed strategies, the micromotors do not require fuel or surfactants for movements and display a high speed of up to 6 mm/s, which assures efficient operation even in complex environments. Although the complete chemical degradation of PS has not been demonstrated, this is not a significant drawback since the capacity of micromotors for the trapping of PS as well as its removal from the medium has been demonstrated. Indeed, while it is true that these micromotors have not been capable of major chemical degradation, it can be said that they have acted as “piranha” micromotors, leaving in their wake deep morphologically damaged and chemically degraded PS particles to some extent. For all these reasons, this stage represents an important advance in demonstrating the potential of this micromotor class in the complex field of MPs elimination. Please note that we chose high-density PS as the model, which is very difficult to degrade in comparison with expanded PS or other models. The new concept illustrated here can be translated to the degradation and removal of other MPs and coupled with additional technologies for further revalorization of the byproducts. Future efforts should be aimed at scaling up the procedure to treat higher water volumes with mixed MPs and to ensure the absence of byproducts in the treated water.

## Supporting Information Available

Supporting figures and tables: SEM characterization of the micromotors, Tauc plots, hydrogen peroxide generation, mechanism for ROS degradation, MALDI-TOF data, and main references related to this work (PDF)
Micromotor swarming and PS particle trapping (Video S1) (MP4)
Characterization of the micromotor/motion (Video S2) (MP4)
Motion control of the micromotors (Video S3) (MP4)
