# Supplementary material for: Photophoretic MoS2–Fe2O3 Piranha Micromotors for Collective Dynamic Microplastics Removal
Source: ACS Appl Mater Interfaces. 2024 Aug 27;16(36):47396–405. doi: 10.1021/acsami.4c06672 (PMC11403556; doi:10.1021/acsami.4c06672)
Supplement: Supplementary file 1 — am4c06672_si_001.pdf [file am4c06672_si_001.pdf]

Supporting information

Photophoretic MoS_2-Fe_2O_3 piranha micromotors for collective dynamic microplastics removal

Víctor de la Asunción-Nadal,[a]‡ *Enrique Solano,*[a]‡ *Beatriz Jurado-Sánchez,*[a,b]* *Alberto Escarpa* [a,b]*

[a]Department of Analytical Chemistry, Physical Chemistry, and Chemical Engineering, Universidad de Alcala, Alcala de Henares, E-28802 Madrid, Spain

[b]Chemical Research Institute "Andres M. Del Río", Universidad de Alcala, Alcala de Henares, E-28802 Madrid, Spain

Corresponding authors. Beatriz Jurado-Sánchez (beatriz.jurado@uah.es) or Alberto Escarpa (alberto.escarpa@uah.es)

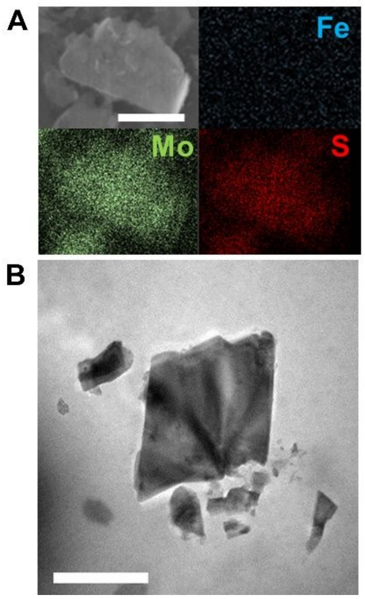

Figure S1. Characterization of the exfoliated MoS_2 micromotors. (A) SEM images and corresponding EDX mapping. (B) TEM images of micromotors.

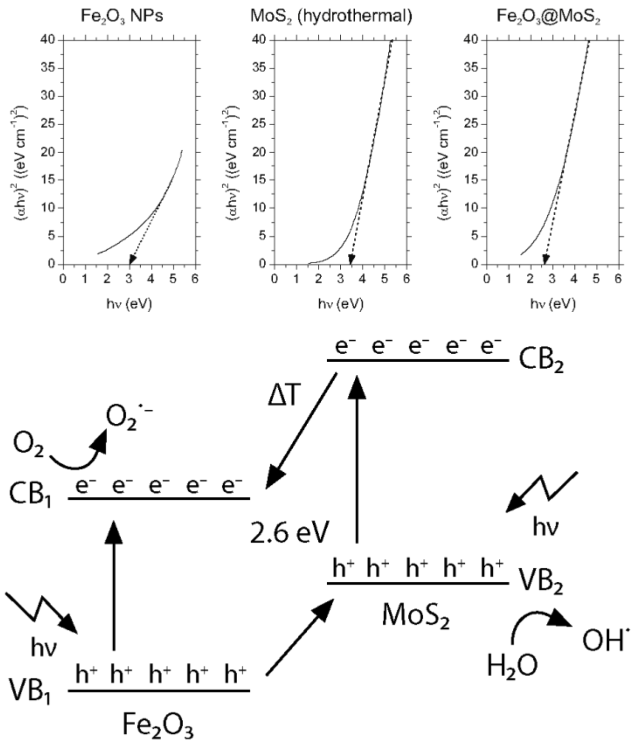

Figure S2. Tauc plots (top) of Fe_2O_3 nanoparticles, MoS_2 synthesized through the hydrothermal method, and Fe_2O_3@MoS_2 micromotors. Schematic of the main processes related to the light-induced generation of ROS and temperature increase.

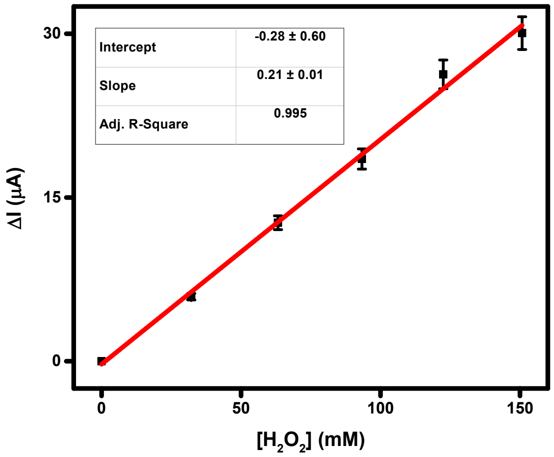

Figure S3 Absolute intensity increments of current under successive H_2O_2 additions.

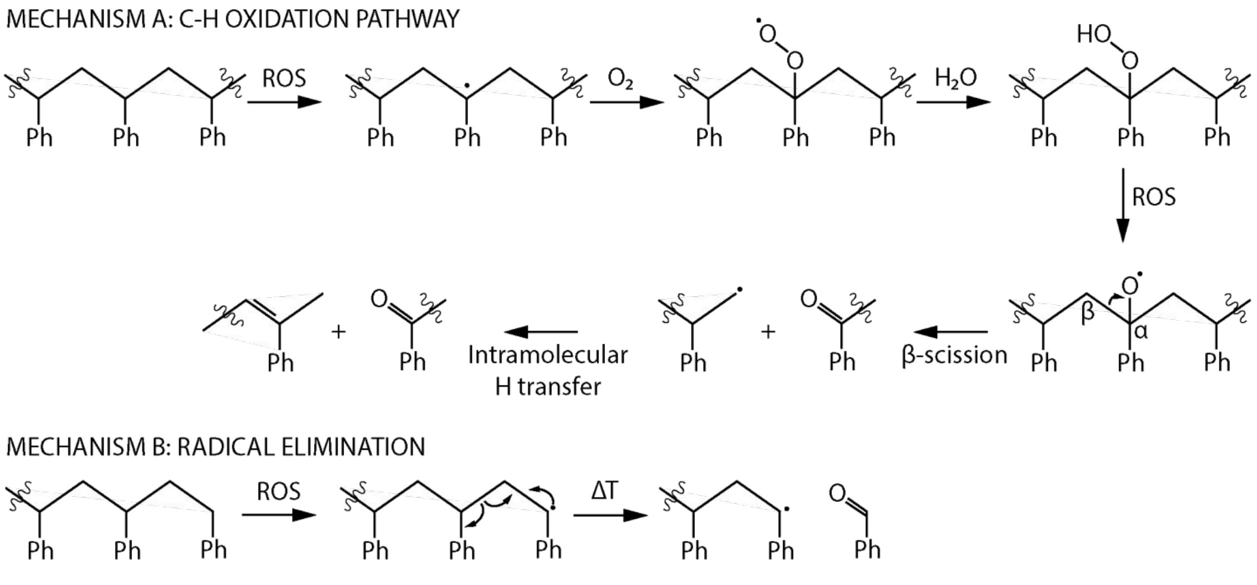

Figure S4. Main reaction mechanisms reported for the ROS-mediated degradation of polystyrene. C-H oxidation pathway (top) and radical elimination (bottom).

Table S1. m/z and relative intensities of the main peaks detected by MALDI-TOF

m/z	Relative intensity (a. u)		
	0 h	2 h	4 h
1199	0.1	0.2	0.2
1330	0.1	-	-
1514	0.3	-	-
1699	0.4	-	0.1
1883	0.3	0.04	0.09
2067	0.1	0.03	0.07
2252	0.05	0.02	0.04

Table S2. Main bibliographical contributions in the field of microplastic remediation using micro/nanomotors.

Microplastic	Micromotor	Mechanism/Action	Reference
Polystyrene	BiOI-Fe_3O_4	Photocatalytic degradation	1
Polystyrene	TiO_2	Photocatalytic degradation	2
Polystyrene	PEGDA-Catalase	Capture	3
Polystyrene	TiO_2	Capture	4
Polyethylene	Fe_2O_3-MnO_2	Catalytic degradation + capture	5

Poly(lactic acid), poly(3-hydroxybutyrate)	Sb_2S_3-Fe_3O_4	Photocatalytic degradation + capture	[6]
Poly(lactic acid), polycaprolactone	$BiVO_4$-Fe_3O_4	Photocatalytic degradation	[7]
Polypropylene	Polydopamine-Fe_3O_4	Enzymatic degradation	[8]
Polystyrene	MoS_2/Fe_2O_3	Photocatalytic degradation + capture + collisions	This work

References

(1) Khairudin, K.; Abu Bakar, N. F.; Osman, M. S. Magnetically Recyclable Flake-Like BiOI-Fe_3O_4 Microswimmers for Fast and Efficient Degradation of Microplastics. *J. Environ. Chem. Eng.* **2022**, *10* (5), 108275. DOI: https://doi.org/10.1016/j.jece.2022.108275.

(2) Chattopadhyay, P.; Ariza-Tarazona, M. C.; Cedillo-González, E. I.; Siligardi, C.; Simmchen, J. Combining Photocatalytic Collection and Degradation of Microplastics Using Self-Asymmetric Pac-Man TiO_2. *Nanoscale* **2023**, *15* (36), 14774-14781, DOI: 10.1039/D3NR01512B.

(3) Ho, H. G. V.; Yoo, P. J. Dual-Catalysts-Embedded Spontaneously Propelling Asymmetric Micromotors Using Triple Emulsion Microfluidic Synthesis for Highly Efficient Nano/Microplastic Removal. *Sep. Pur. Technol.* **2024**, *351*, 127952. DOI: https://doi.org/10.1016/j.seppur.2024.127952.

(4) Ullattil, S. G.; Pumera, M. Light-Powered Self-Adaptive Mesostructured Microrobots for Simultaneous Microplastics Trapping and Fragmentation via in situ Surface Morphing. *Small* **2023**, *19* (38), 2301467. DOI: https://doi.org/10.1002/smll.202301467.

(5) Ye, H.; Wang, Y.; Liu, X.; Xu, D.; Yuan, H.; Sun, H.; Wang, S.; Ma, X. Magnetically steerable Iron Oxides-Manganese Dioxide Core–Shell Micromotors for Organic and Microplastic Removals. *J. Coll. Interface Sci.* **2021**, *588*, 510-521. DOI: https://doi.org/10.1016/j.jcis.2020.12.097.

(6) Jancik-Prochazkova, A.; Jašek, V.; Figalla, S.; Pumera, M. Photocatalytic Microplastics "On-The-fly" Degradation via Motile Quantum Materials-Based Microrobots. *Adv. Optical Mater.* **2023**, *11* (22), 2300782. DOI: https://doi.org/10.1002/adom.202300782.

(7) Beladi-Mousavi, S. M.; Hermanová, S.; Ying, Y.; Plutnar, J.; Pumera, M. A Maze in Plastic Wastes: Autonomous Motile Photocatalytic Microrobots against Microplastics. *ACS Appl. Mater. Interfaces* **2021**, *13* (21), 25102-25110. DOI: 10.1021/acsami.1c04559.

(8) Zhou, H.; Mayorga-Martinez, C. C.; Pumera, M. Microplastic Removal and Degradation by Mussel-Inspired Adhesive Magnetic/Enzymatic Microrobots. *Small Meth.* **2021**, *5* (9), 2100230. DOI: https://doi.org/10.1002/smtd.202100230.

(
